# Infliximab-Induced Improvement in Dercum's Disease

**DOI:** 10.7759/cureus.61499

**Published:** 2024-06-01

**Authors:** Cailean E McKay, Ishaan Batish, Shiva Arami

**Affiliations:** 1 Internal Medicine-Pediatrics, University of Illinois Chicago, Chicago, USA; 2 Internal Medicine, Sri Guru Ramdas Institute of Medical Sciences and Research, Amritsar, IND; 3 Rheumatology, University of Illinois Chicago, Chicago, USA

**Keywords:** tnf inhibitors, methotrexate, infliximab, pain control, painful lipomas, dercum’s disease

## Abstract

Dercum’s disease (DD) is a rare and poorly understood disease characterized by obesity and painful lipomas throughout the body. Although the entity is well described in the literature, its etiology, prevalence, and treatment remain unclear. Currently, treatment is focused on pain management. We describe a case of a patient with DD who showed improvement with infliximab and methotrexate.

## Introduction

Dercum’s disease (DD), also called adiposis dolorosa, is a rare entity that is characterized by obesity and chronic pain due to subcutaneous adipose tissue growth, predominantly seen in women aged 35-50 [1]. Often seen additional symptoms include weakness, fatigue, easy bruising, sleep disturbances, dyspnea, joint pain, and neuropsychiatric symptoms, such as emotional instability and depression [2]. The painful adipose tissue can be diffuse or localized to lipoma-like masses. The most commonly affected areas are the extremities, trunk, pelvic area, and buttocks.

DD is a diagnosis of exclusion and is made clinically. On pathology, excised tissue is consistent with fatty connective tissue without signs of inflammation [3]. On examination and imaging, the tender tissue often appears similar to a lipoma, and there are no laboratory tests to distinguish between the two [4].

There is currently no standard treatment for DD, and effective treatment is lacking. Primarily, management has been through pain control, both through medication and surgical excision of painful lesions [5-7]. Patient’s with DD are often prescribed nonsteroidal anti-inflammatory drugs (NSAIDs) and various formulations of lidocaine. There have been various case reports showing the benefit of a variety of different agents [8-11], as well as a single case report showing improvement with infliximab and methotrexate [12].

## Case presentation

A 56-year-old female was referred to our clinic in 2019 for further evaluation of DD. She had received a diagnosis two years prior, following a prolonged period of developing painful soft tissue nodules, predominantly in her legs. During this time, she underwent multiple surgeries to remove approximately 30 of these masses.

Past medical history was significant for obesity (body mass index (BMI) > 40), type 2 diabetes mellitus, fibromyalgia, hypertension, and possibly Crohn’s disease. Review of records from her prior rheumatologist showed mildly elevated sedimentation rate, negative rheumatoid factor, and ANA positive at a titer of 1:40. For her DD, she had tried pregabalin, gabapentin, NSAIDs, duloxetine, topiramate, and opiates with little relief or intolerable side effects. Her sleep and activities of daily living (ADLs) were impacted by the level of pain she experienced. She was able to tolerate tramadol, which was prescribed to her by a pain management clinic. Eventually, methotrexate was initiated at 25 mg subcutaneously weekly. She did experience some improvement with the methotrexate, but eventually felt like her pain was worsening again, at which point she was referred to our clinic.

Given her previous improvement with methotrexate, infliximab was initiated at a dose of 3 mg/kg, administered every eight weeks after loading doses. After four infusions, she began to report improvement in her pain. After several months, improvement in size of some of her fatty nodules were noted on in-clinic ultrasound.

Unfortunately, after eight months of infliximab, the patient was unable to return to the clinic for infusions due to the COVID-19 pandemic. In May 2020, approximately six months after her last infusion, she returned to the clinic and reported significantly increased pain. She was unable to restart infliximab until February 2021 due to surgery to remove several fatty nodules. In the interim, she established with a pain clinic and used Norco 10 mg every four hours as needed for pain. Infliximab was restarted and her dose was increased to 5 mg/kg, as well as restarting methotrexate at a dose of 10 mg weekly. She initially felt significant improvement with reinitiation of infliximab. However, by July 2021, the patient no longer felt like she was experiencing improvement, so both infliximab and methotrexate were discontinued. The patient was lost to follow-up until May 2022; in the interim, she had surgery to remove several painful lipomas. At that time, she was discharged from our rheumatology clinic and instructed to follow with pain management.

The patient's timeline with our clinic is summarized in Table 1.

**Table 1:** Timeline of the patient presentation, symptoms, and treatment

Date	Clinical activity
March 2019	First visit to the clinic
April 2019	First infliximab infusion at 3 mg/kg
June–December 2019	Infliximab infusions every eight weeks; pain improvement noted during clinic visits
January 2020	Improvement in the size of forearm lipomas noted on ultrasound
February–May 2020	No infliximab due to the COVID-19 pandemic
May 2020	Telehealth visit, endorsed worsening pain since stopping infliximab
August 2020	Surgery to resect lipomas, clinic visit
September 2020	Established with pain clinic
January 2021	Clinic visit, decision made to restart infliximab and methotrexate
February 2021	First infliximab infusion at 5 mg/kg
May 2021	Clinic visit with reported improvement in pain
July 2021	Experiencing significant pain, requested to stop infliximab and methotrexate
April 2022	Returned to clinic for refill of pain medication, instructed to follow up with pain clinic

## Discussion

**Figure 1:**
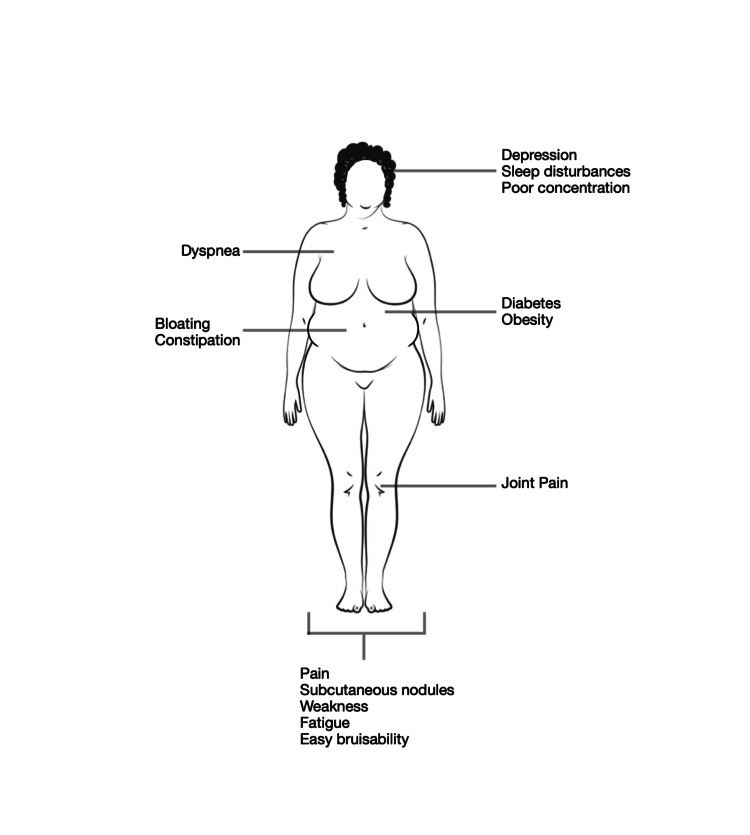
Signs and symptoms of Dercum's disease Adapted from Hansson et al [2]. This is an Open Access article distributed under the terms of the Creative Commons Attribution License.

DD, also known as adiposis dolorosa, is a rare disorder characterized by painful subcutaneous lipomas most frequently distributed on the extremities, trunk, and buttocks. Associated symptoms include weakness, fatigue, easy bruisability, sleep disturbances, joint pain, and neuropsychiatric complaints, such as depression and poor concentration (Figure 1) [3].

It has been proposed that DD be defined as chronic (>3 months) painful adipose tissue in conjunction with obesity. Four distinct subtypes have been proposed: type I, a generalized diffuse form with widespread, painful fatty tissue without any distinct lipomas; type II, a generalized nodular form presenting with widespread pain in and around multiple lipomas throughout the body; type III, a localized nodular form with painful lipomas in limited areas; and type IV, the juxta-articular form that presents with painful fatty tissue near large joints, such as the knee, hip or elbow, as summarized in Table 2 [2]. Most cases of DD appear to occur sporadically, although there have been reports suggesting an autosomal dominant inheritance pattern [13].

**Table 2:** Four subtypes of Dercum's disease

Type	Description	
I	Generalized diffuse	Diffusely widespread painful adipose tissue without clear lipomas
II	Generalized nodular	General pain in adipose tissue and intense pain in and around multiple lipomas
III	Localized nodular	Pain in and around multiple lipomas
IV	Juxta-articular	Solitary deposits of excess fat, for example at the medial aspect of the knee

The pathogenesis of DD has not been elucidated, although various theories have been proposed. Compression of nerves by adipose tissue and disturbance of subcutaneous blood flow are common theories [14], but this has never been shown on pathology [2-4]. Endocrine dysfunction or alterations in lipid metabolism have also been suggested, given the prevalence of metabolic derangements, such as diabetes in patients with DD [1]. By contrast, it has also been shown that adipocytes secrete pro-inflammatory cytokines, including TNF-alpha and interleukin-6 [15].

Given that the underlying mechanism of DD is not known, there is no standard treatment and therapy has mainly focused on pain relief. Liposuction is a fairly common surgical method used to treat areas of painful fat [3]. Several small studies have shown improvement in pain after these procedures, although the pain relief may diminish over time [16]. However, liposuction is more effective for patients with diffuse disease, rather than a nodular form. Dermolipectomy or lipectomy have also been used for larger, nodular masses [5]. Transcutaneous electrical stimulation has been reported as a safe and effective treatment, and a small study has shown whole body pneumatic compression to be effective [3].

Pharmacologically, treatment in the past has focused mostly on pain relief. Lidocaine has been used in the form of topical application, intralesional injections, and infusions. The latter two forms have been shown to bring relief for multiple months [6,7]. There are mixed reports on corticosteroids; generally, they provide no relief, and one case study showed a link between corticosteroid use developing DD, although conversely, one case report has shown improvement with intralesional injections [3]. Recently, it has been reported that injections of deoxycholic acid into lipomas of patients with DD improved pain [3]. Oral NSAIDs and opiates have been a mainstay for pain relief, and there have been cases showing benefits with metformin [8], pregabalin [9], interferon alpha-2b [10], and d-amphetamine [11].

Previously, there had been one case report showing improvement with methotrexate and infliximab in a patient with DD who also had a history of ankylosing spondylitis [12]. Given the secretion of TNF-alpha and IL-6 by adipocytes [17], there is a reason to believe that anti-TNF therapy would lead to improvement in pain and inflammation. Furthermore, TNF-alpha has been implicated in the release of free fatty acids [15], leading to a decrease in peripheral insulin sensitivity; if fatty acid metabolism plays a role in DD, inhibition of this process could lead to improvement. In our case, the patient did show improvement with her initial course of infliximab, and some improvement with re-initiation. Given the well-known fact that patients can develop antibodies to TNF inhibitors [18], it is possible that her lack of long-term improvement with infliximab was secondary to development of these antibodies, given the intermittent nature of her treatment. TNF-inhibitors, however, do have risk of serious but rare side effects, including invasive fungal infections and tuberculosis [19,20], so they must be used with caution.

## Conclusions

DD is a rare condition characterized by painful lipomas, obesity, and nonspecific constitutional symptoms. Patients with DD are difficult to diagnose and manage, given the rarity of its presentation and the lack of standardized treatment. This case report brings further attention to methotrexate and infliximab as a possible treatment for patients with refractory disease. Further research is necessary to help find a definitive method for diagnosing and treatment of DD and to determine etiology.
